# An Account of the Effects of a Large Dose of Saccharum Saturni

**Published:** 1783

**Authors:** 


II. *An Account of the effects of a large dofe of Saccharum Saturni. By Mr.* Francis Knight, *Surgeon of the Coldftream regiment of Foot-guards. Communicated in a letter to Dr.* Saunders, *phyfician to Guy's Hofpital, and by him to Dr.* Simmons.

JOHN Grazebrook, a recruit in the Coldftream regiment of foot-guards (having contracted a recent gonorrhoea which he was unwilling to make known to the regiment) took by the advice of a friend a medicine, which fince proves to have been full two drachms of *Sacch. Saturn.* mixed with milk.

On Thurfday night, June 5th, he took half of this quantity, and in about five hours after, complained of great pain in his bowels and back, with a very violent and painful diftenfion above and below the navel; but, not fufpecting his illnefs to have been occafioned by the medicine, he he took the remaining part of it. Within an hour his complaints greatly increaſed upon him, he totally loſt the power of ſpeech, and afterwards his ſenſes. In this ſtate, and apparently in great pain, he was brought the next morning to the regimental hoſpital. He had moſt profuſe and general perſpiration, his pulſe was uncommonly ſoft and ſlow (not exceeding 40 in a minute) and he was frequently vomiting up a green bilious fluid. We could gain no certain information of what he had taken; but, from ſome ſuſpicion of the poiſon, he was freely drenched with warm water and oil. The ſame was thrown up by way of clyſter, and being often repeated, with purgative medicines by the mouth, his bowels were with ſome difficulty opened.—After this, late on the Friday evening, he appeared ſomewhat ſenſible, but did not in the leaſt recover his ſpeech till the following day (June 7th); when in a faultering voice he aſked for ſome liquor. He complained of a pain in his head, but was pretty eaſy in his bowels, having had eight or nine ſtools. His perſpiration continued all this time equally profuſe and general; but about four o'clock on the Sunday morning it was ſomewhat interrupted by a cold ſhivering of half an hour's continuance, ſucceeded ceeded by heat, and which terminated again in a profuse perspiration. This continued about two days more, his voice becoming gradually more distinct, and his bowels keeping regularly open without the aid of medicine. His pulse was still very slow, not beating more than 56 or 58 (even after meals) but he had no farther complaint in his bowels, nor any subsequent paralytic symptom. He continued mending till the 13th, when he was discharged the hospital, free of all complaints, and fit for duty.

It is not unworthy of remark, that his clap (which, in two days time, had stained the whole flap of his shirt with a most virulent discharge) was in nearly the same space of time perfectly cured. I examined his urethra on the Sunday (June 8th) but could not press out the least drop of running. The mouth of the urethra betrayed no appearance of inflammation, nor had he any return of it before he left the hospital. —It is still more extraordinary (though equally true) that some old venereal chancres under the prepuce, which had withstood a pretty long course of mercury from the surgeon of the regiment he before belonged to, seemed as suddenly to have assumed a healing appearance.

